# Morel-Lavallée Lesions: A Diagnostic and Clinical Dilemma

**DOI:** 10.7759/cureus.28038

**Published:** 2022-08-15

**Authors:** Paul Yager, Robert M Chory, Austin Dennis

**Affiliations:** 1 Radiology, Grandview Medical Center, Birmingham, USA; 2 Obstetrics and Gynecology, Edward Via College of Osteopathic Medicine (VCOM), Auburn, USA

**Keywords:** degloving injury, closed degloving, lower extremity trauma, morel-lavallée, morel-lavallée lesions

## Abstract

Morel-Lavallée lesions (MLL) occur following closed degloving injuries in trauma and appear as subcutaneous fluid collections. These lesions provide a diagnostic dilemma due to their resemblance to other subcutaneous lesions, including post-traumatic or post-procedural hematomas, and malignant entities such as soft tissue sarcomas. Magnetic resonance imaging (MRI) remains the diagnostic test of choice to diagnose MLL. Here we present a single case of MLL in a 40-year-old female who sustained an injury to her left thigh months earlier after falling through a pier as well as a review of literature on the diagnostic findings typically seen in this type of injury.

## Introduction

Morel-Lavallée lesions were first described in 1863 by Victor Auguste Francois as a subcutaneous fluid collection. In recent literature, MLL has been described as a closed degloving injury, usually caused by direct trauma due to motor vehicle accidents (MVA), but also seen in other high-energy trauma, contact sports, and more recently abdominoplasty [1]. The inciting event results in detachment of the subcutaneous tissues from the deep fascia allowing for serosanguinous fluid to fill the pathologic dead space. The vast majority of these lesions occur in adults, with the most common region being the upper thigh. However, they can be seen in children with the earliest reported case being a 28-month-old child with left gluteal MLL following an MVA [2].

MLL lesions have a variable incidence, with reported numbers ranging from 10 cases per year (in a trauma center) to 8.3% of all pelvic trauma cases in another series [1-3]. Unsurprisingly, males are predominantly affected, similar to the increased incidence of trauma in males. The most commonly involved region is the upper thigh, followed by the soft tissue of the pelvis and knee. These locations are postulated to be affected at a higher rate due to the large tissue tension gradient between the fascial planes and the subcutaneous tissue [1,3].

## Case presentation

A 40-year-old woman with no prior significant medical history presented for evaluation of pain in her right thigh. She reported falling through a rotted pier several months prior to the onset of pain. She stated the pain was minimal after the incident but progressively worsened with redness and tenderness being maximal on the day of examination. On examination, the patient had tenderness, edema, and erythema of the right lateral thigh. The patient was afebrile and had no signs concerning for cellulitis at this time. The patient denied pain in the contralateral leg or feet bilaterally. No obvious fractures or crepitus were felt on palpation. Achilles and patella reflexes were both 3/4 bilaterally, and sensation was intact throughout each leg. Bilateral pedal and posterior tibial pulses were normal.

**Figure 1:**
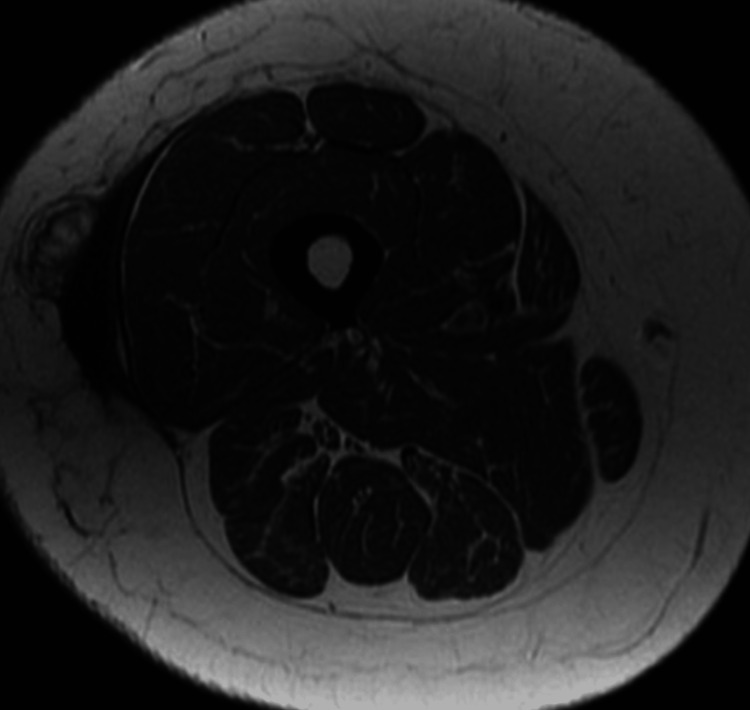
T1-weighted axial MRI through the right mid-thigh showing a hypointense collection in the right lateral thigh.

**Figure 2:**
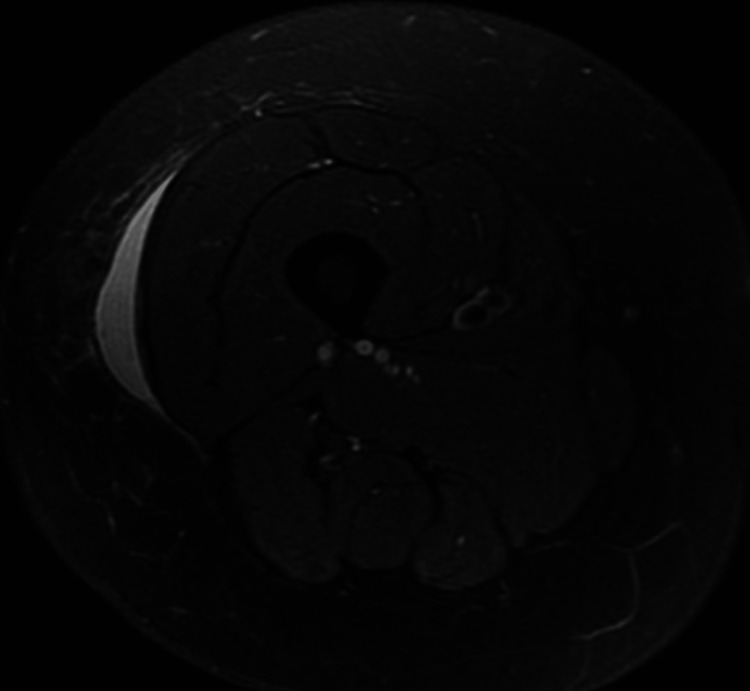
T2-weighted axial MRI through the right thigh showing a simple, hyperintense fluid collection.

**Figure 3:**
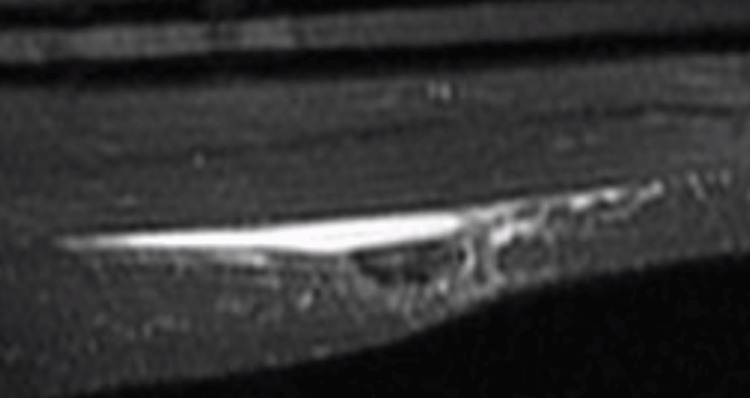
T2-weighted coronal MRI through the right thigh demonstrating fluid showing subcutaneous tissue and vastus lateralis.

A non-contrast MRI was ordered showing a subcutaneous fluid collection in the right lateral thigh containing about 100 mL of fluid (Figures 1-3). Fine needle aspiration of the mass was performed showing bland fluid. Fluid analysis was not significant for increased leukocytes or bacteria. The patient reported relief of pain after fine needle aspiration and has not shown recurrence of the lesion at follow up appointments.

## Discussion

Pathology and presentation

The formation of the potential space in MLL is most commonly triggered by trauma. This subcutaneous pseudo-lumen allows serous fluid, fat, or other debris to collect. The high mobility of joints like the hip and knee, paired with the tough underlying fascia results in a form of closed degloving lesion. MLL should be considered in any patient who presents with a painful, enlarging subcutaneous fluid collection secondary to trauma [1,4]. This is especially true for collections that present in a delayed fashion or do not resolve after a normal time period; if the patient has resolving hematomas or surgical seromas, these may be used as a guide to differentiate a persistent MLL. While motor vehicle accidents are the most commonly reported trigger for the formation of MLL, other entities have become increasingly common in literature [1]. These include sports injuries and abdominoplasties [1,3,4].

Clinical presentation varies but typically involves a painful and swollen mass. Other reported symptoms include decreased sensation due to compression of cutaneous nerves, skin discoloration, and subcutaneous crackling. Given that trauma is the most common cause of MLL, the clinical presentation is usually overshadowed by the injuries from the insulting event [3]. The MLL can mimic many other forms of trauma-related masses such as hematomas, post-operative seromas, and fat necrosis; this likely contributes to the wide range of incidence reported in literature [1,4]. Other mimics include hemangiomas or bursitis; a potentially fatal doppelganger includes soft tissue sarcomas and abscesses. The amount of overlap between presenting symptoms and findings on imaging makes diagnosing MLL difficult [1,4].

Treatment and complications

The classification of MLL is currently limited to the Mellado and Bencardino classification system. This stratifies lesions based on MRI characteristics such as presence of a capsule. However, this classification scheme fails to provide any correlation between the type of lesion and the prognosis or management. A more recent scheme, suggested by Singh et al., takes into account chronicity of the lesion and lesion size, to stratify the lesions into conservative care (compressive bandage), drainage with sclerodesis, or open surgical management [4]. Other important considerations which have not yet made it into a classification system include stability of the patient, location of the lesion, and the presence of any other concomitant injuries sustained from the inciting traumatic incident. Compression has been more successful in smaller acute lesions that lack a capsule. Aspiration alone results in a high recurrence rate, whereas sclerodesis is more effective in chronic lesions containing up to 700 mL of fluid [1-3]. Sclerodesis ultimately results in granulation tissue formation and fibrosis of the capsule. Surgical resection is usually reserved for large lesions with an extensive capsule. The rarity of MLL and lack of information on management is an area that requires more research.

The majority of complications from MLL result from late diagnosis or misdiagnosis. Late diagnosis can result in an expansive lesion causing pressure necrosis or compartment syndrome. Long-standing fluid collections can organize into infection [1,4]. Late diagnosis is also associated with formation of a capsule after the fluid contained within the cavity has been resorbed, ultimately resulting in a chronic MLL, persistent inflammation, and increasing the need for surgical treatment of the lesion. Misdiagnosis and subsequent surgery performed through the lesion for concomitant traumatic injuries can result in a higher rate of infection [1,4].

## Conclusions

Although Morel-Lavalée lesions are not a new phenomenon, these rare-to-nearly-common, closed degloving injures present both a clinical and diagnostic dilemma as there are many other conditions that present similarly. With a broad differential diagnosis, no standard approach to treatment, and numerous complications that have the potential for significant morbidity, MLL deserves careful consideration. We have presented a single case of a 40-year-old woman who had substantial trauma to the left lower extremity resulting in MLL of the left upper thigh. She underwent treatment with aspiration and compression and is clinically doing well. In the future, awareness of this lesion, development of a stratifying classification scheme, and standardization of treatment would serve to improve patient outcomes.

## References

[1] Volavc TS, Rupreht M (2021). MRI of the Morel-Lavallée lesion - a case series. Radiol Oncol.

[2] Rha EY, Kim DH, Kwon H, Jung SN (2013). Morel-Lavallee lesion in children. World J Emerg Surg.

[3] Rashid A, Singh MK, Feng SS, Yatim NM, Sahak MY, Mahmud R (2020). Lethal Morel-Lavallée lesion: a forensic radiology-pathology correlation. Radiol Case Rep.

[4] Singh R, Rymer B, Youssef B, Lim J (2018). The Morel-Lavallée lesion and its management: a review of the literature. J Orthop.

